# Supplementary material for: Analysis and Comparison of Rapid Methods for the Determination of Ochratoxin a Levels in Organs and Body Fluids Obtained from Exposed Mice
Source: Toxins (Basel). 2022 Sep 13;14(9):634. doi: 10.3390/toxins14090634 (PMC9503121; doi:10.3390/toxins14090634)
Supplement: Supplementary file 1 [file toxins-14-00634-s001.zip › toxins-1874636-supplementary.pdf]

toxins

MDPI

Article

Analysis and Comparison of Rapid Methods for the Determination of Ochratoxin A Levels in Organs and Body Fluids Obtained from Exposed Mice

Zsuzsanna Szőke, Bianka Babarczi, Miklós Mézes, István Lakatos, Miklós Poór, Eszter Fliszár-Nyúl, Miklós Oldal, Árpád Czéh, Kornélia Bodó, György Nagyéri and Szilamér Ferenczi

Table S1. The weight of the body and tissues examined.

Treatments	Initial BM	Final BM	AVE BM (g)	AVE kidney (g)	AVE norm. kidney (g)	AVE spleen (g)	AVE norm. spleen (g)
OTA-1	43.0 ± 1.34	41,2 ± 1.25	40 ± 1.24	0,657 ± 0.051	0,02 ±0 .001	0,1230 ± 0.006	0,0029 ± 0.0001
OTA-10	41.0 ± 0.71	38,5 ± 0.82	42.5 ± 0.77	0,586 ± 0.030	0,01 ± 0.004	0,1400 ± 0.014	0,0036 ± 0.0004
VC	39,8 ± 1.65	39 ± 1.46	39,3 ± 1.08	0,638 ± 0.053	0,02 ± 0.005	0,1296 ± 0.013	0,0033 ± 0.0002

* the initial and final body mass (BM), average (AVE), and normalized (norm.) parameters of kidney and spleen.

Table S2. Linear regression: interpretation of Sy.x values.

Blood/organs	Comparison of methods	*Sy.x*
blood	HPLC *vs.* ELISA	1.351
	HPLC *vs.* FCM	1.614
	FCM *vs.* ELISA	2.362
liver	HPLC *vs.* ELISA	0.3193
	HPLC *vs.* FCM	0.1484
	FCM *vs.* ELISA	0.4263
kidney	HPLC *vs.* ELISA	0.3714
	HPLC *vs.* FCM	0.0909
	FCM *vs.* ELISA	0.3906

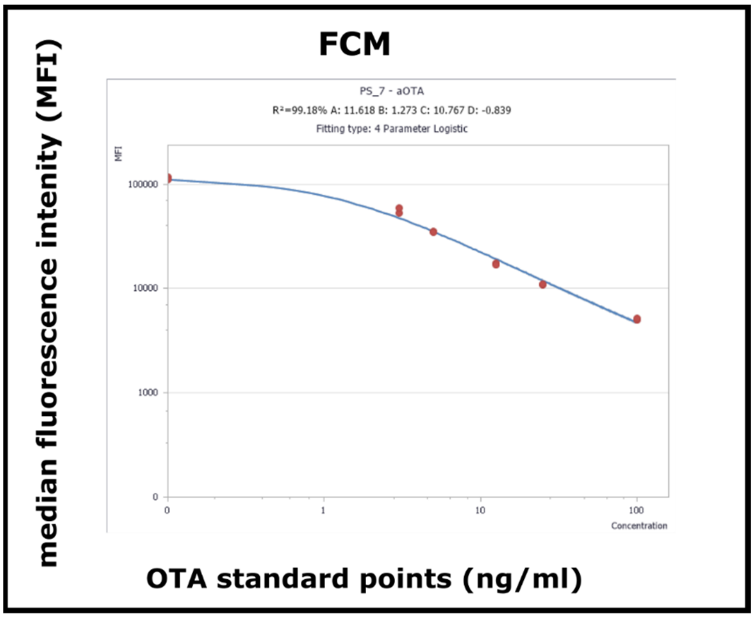

Figure S1. Representative standard curve from one FCM measurement. OTA standard points cover the entire range from 0 to 100 ng/ml concentration. In addition, a competitive arrangement was applied, where the OTA concentrations (ng/ml) were inversely proportional to the median fluorescence intensity (MFI). 4 parametric logistic curves were fitted to OTA standard points to evaluate the exact OTA concentration in samples.

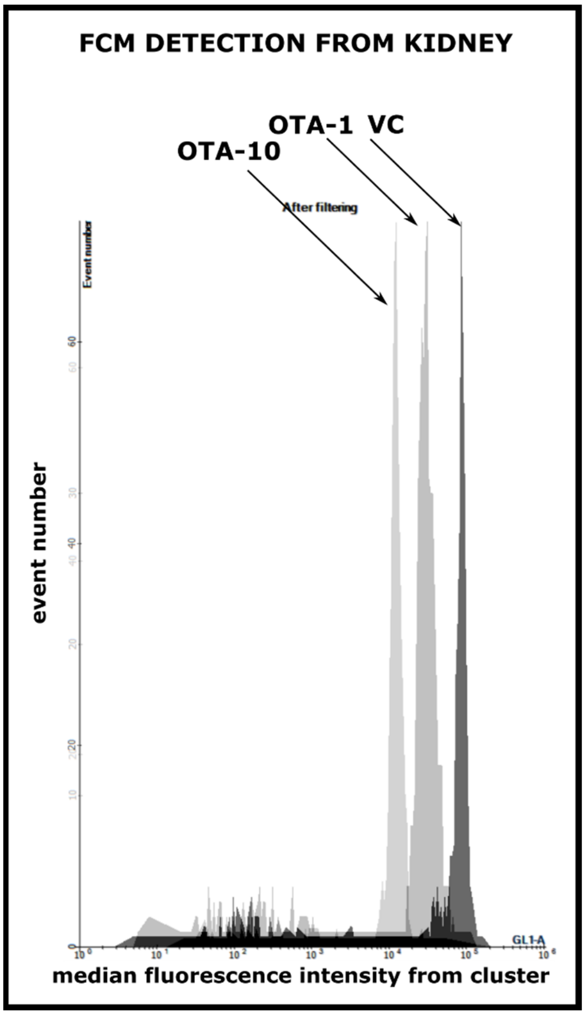

Figure S2. Representative histograms of one FCM measurement using kidney samples. Peaks can be distinguishable by their median fluorescence intensity. VC, OTA-1, and OTA-10 groups formed separate, well-distinct clusters because the OTA-content of samples could interfere with the fluorescent OTA-conjugate as a tracer. Increasing OTA content in samples resulted in a “shift” in the median fluorescence intensity, which thus appeared as discrete peaks.

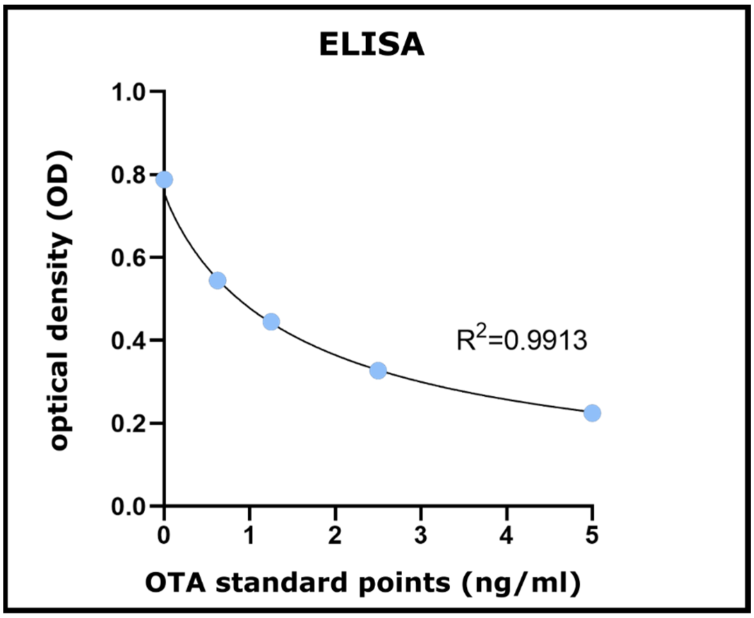

Figure S3. Representative standard curve of one ELISA measurement using blood plasma samples. The size of ODs is inversely proportional to the OTA concentration used for standards. Four parametric logistic curves were fitted to the OTA standard points to calculate exact OTA concentrations in samples.

2.4.

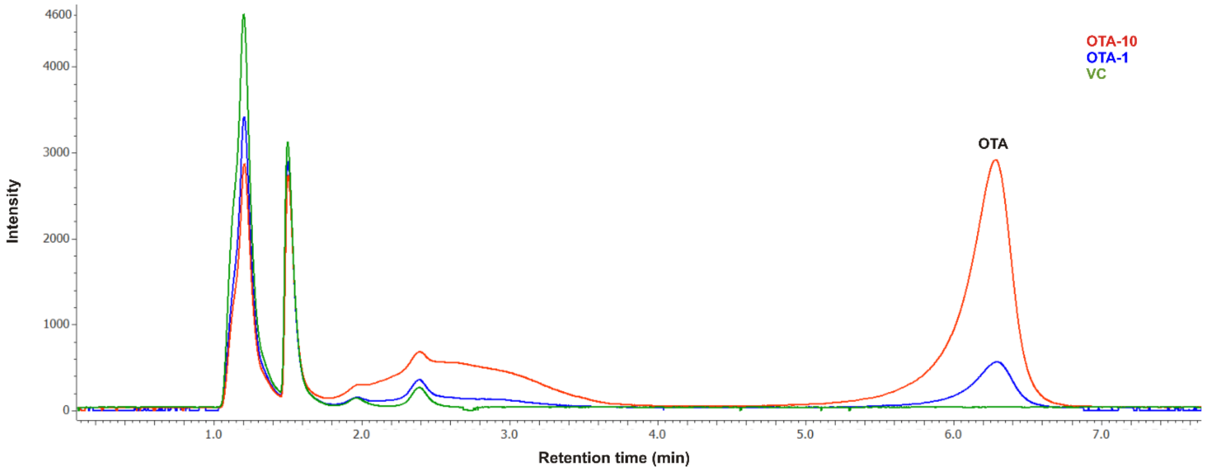

Figure S4. Representative chromatograms of HPLC-FLD measurements from the kidney samples of OTA-exposed mice and control animals.
